# A systematic review of prevention interventions to reduce prenatal alcohol exposure and fetal alcohol spectrum disorder in indigenous communities

**DOI:** 10.1186/s12889-018-6139-5

**Published:** 2018-11-03

**Authors:** Martyn Symons, Rebecca Anne Pedruzzi, Kaashifah Bruce, Elizabeth Milne

**Affiliations:** 1 Telethon Kids Institute, The University of Western Australia, PO Box 855, West Perth, Perth, WA 6872 Australia; 2 National Health and Medical Research Council FASD Research Australia Centre of Research Excellence, Perth, Australia

**Keywords:** FASD, FAS, Aboriginal, Indigenous, Alcohol, Pregnancy, Prevention

## Abstract

**Background:**

Fetal alcohol spectrum disorder (FASD) is a preventable, lifelong neurodevelopmental disorder caused by prenatal alcohol exposure. FASD negatively impacts individual Indigenous communities around the world. Although many prevention interventions have been developed and implemented, they have not been adequately evaluated. This systematic review updates the evidence for the effectiveness of FASD prevention interventions in Indigenous/Aboriginal populations internationally, and in specific populations in North America and New Zealand, and offers recommendations for future work.

**Method:**

The MEDLINE, Embase, CINAHL Plus, Web of Science, PsycINFO, SocINDEX, and Informit databases were searched from inception to 22/08/2017 for all prevention and intervention papers published in peer-reviewed scientific journals, with results, targeting prenatal alcohol exposure and FASD in Indigenous populations. This review was limited to studies published in English and excluded interventions focusing on the workforce. All steps were completed independently by two reviewers with discrepancies resolved via consensus with the senior author.

**Results:**

There was significant heterogeneity in the ten included studies. Populations targeted included non-pregnant women of child-bearing age, pregnant women, school children and the general public. Study designs included one randomised controlled trial, five cohort studies with pre-post design, one cross-sectional study with different pre- and post-intervention groups, and four studies collected post-intervention data. Studies assessed changes in knowledge, and/or changes in risk for prenatal alcohol exposure including self-reported alcohol consumption, use of birth control or a combination of both. One study was conducted in Australia and nine in the US. The methodological quality of all studies was rated as ‘Poor’ using the systematic review assessment tools developed by The National Heart, Lung and Blood Institute. Studies were subject to substantial bias due to issues such as high loss to follow-up, lack of control groups and the reliance on self-report measures to assess the main outcome.

**Conclusion:**

Overall, there is little evidence that previous interventions aiming to reduce the risk of prenatal alcohol exposure or FASD in Indigenous populations have been effective. Future intervention studies should address the cultural factors and historical context that are fundamental to successful work with Indigenous populations, and be designed, implemented and evaluated using rigorous methods.

This systematic review was registered with PROSPERO, CRD42018086212.

**Electronic supplementary material:**

The online version of this article (10.1186/s12889-018-6139-5) contains supplementary material, which is available to authorized users.

## Background

Fetal Alcohol Spectrum Disorder (FASD) is a term used to describe a range of lifelong, severe disabilities associated with fetal exposure to alcohol. Fetal Alcohol Syndrome (FAS), also now known as ‘FASD with three sentinel facial features’ in Australia, is part of the FASD spectrum and represents a small proportion of FASD cases. People born with FASD experience severe neurodevelopmental impairments such as poor memory and cognitive skills, learning difficulties, and diminished ability to regulate behaviour and affect [1]. There are many health and psychosocial consequences of FASD [2] such as disrupted education, difficulties gaining employment, mental health and substance abuse problems, and engagement with the justice system. The economic burden associated with FASD is significant, and impacts society across individual, family and community levels [3].

The prevalence of FASD is difficult to ascertain, and underestimates are likely for several reasons. Diagnosis is complex, time-consuming and costly, requiring each child to undergo comprehensive physical, developmental and psychometric assessments with a multidisciplinary team (Paediatrician, Psychologist, Speech Pathologist, Occupational Therapist), typically led by a medical specialist [4]. Many cases go undiagnosed, due to lack of awareness of the condition amongst health professionals, and limited availability of diagnostic services [5]. This is especially true in rural and remote areas where access to expert assessment is further obstructed by geographical distance.

A recent meta-analysis estimated the global prevalence of FASD in the general population of children and youths (ages 0–16.4) to be 7.7 per 1000 [6]. In some countries or regions, its burden is disproportionately borne by Aboriginal peoples. For example, the estimated prevalence of FASD in Canadian Aboriginal people is 86.8 per 1000, compared with 5.3 per 1000 in the general population (all ages) [7]. The same review reported the prevalence among Aboriginal people in the United States (US) to be 9.5 per 1000, compared with 15.2 in the general population (all ages). The authors considered this unlikely to be a true representation given that alcohol consumption during pregnancy was almost three times higher in Aboriginal women and the prevalence of FAS was higher in Aboriginal people (3.8 per 1000) than the general population (2.3 per 1000). The prevalence of FASD diagnosed in children up to 6 years of age and notified to the Western Australian Register of Developmental Anomalies was over 130 times more common in Aboriginal (4.08 per 1000) compared with non-Aboriginal births (0.03 per 1000) although both are likely to be underestimates [8]. A national study through the Australian Paediatric Surveillance Unit reported that 65% of FAS cases diagnosed by 15 years of age by practicing paediatricians using active case ascertainment over 4 years were born to mothers identifying as Aboriginal or Torres Strait Islander [9]. Finally, the Lililwan project, conducted in a remote Australian Aboriginal Community, is the only active case ascertainment study undertaken in Australia. The prevalence of FASD in this cohort of 7–8 year-old children was estimated to be 194.4 per 1000 [10]. Understanding risk factors for prenatal alcohol exposure (PAE) and identifying successful programs that can reliably reduce the likelihood of PAE amongst Indigenous people is therefore of utmost importance.

There is currently no known safe level of alcohol consumption during pregnancy [11], and the Australian Government advises that “For women who are pregnant or planning a pregnancy, not drinking is the safest option.” (https://www.nhmrc.gov.au/health-advice/alcohol, accessed 21/12/2017). Similar guidelines recommending abstaining from alcohol during pregnancy exist in North America. Nevertheless, drinking during pregnancy is relatively common ranging from 35.6 to 58.7% in Australia [12, 13] and 10% to 15% in Canada and the US [7]. The prevalence of alcohol use during pregnancy has been estimated to be considerably higher in Aboriginal populations in North America (Canada 36.5% and USA 42.9%), including binge drinking (Canada 22.1% and US 14.6%) [7]. Corresponding estimates for Indigenous populations in Australia are not available. Alcohol consumption before pregnancy recognition is also a problem, as unplanned pregnancies are relatively common. For example, over 47% of pregnancies are unplanned in North America [14] and over 40% of Australia women have experienced an unplanned pregnancy at some time [15]. As a consequence, 60% of Australian women drank alcohol between conception and pregnancy recognition [16].

As FASD is caused by alcohol exposure during pregnancy, it is preventable. Interventions designed to prevent PAE, and therefore FASD, have taken many forms. A previous systematic review [17] identified three broad categories of FASD prevention strategies. The *universal* approach aims to educate the general public about the risks associated with alcohol use during pregnancy. S*elective* approaches target women of childbearing age and their partners who are at risk of having a child with FASD because they consume alcohol and/or belong to a vulnerable subgroup. *Indicated* strategies specifically target women who have previously consumed alcohol at risky levels during pregnancy, had a child with FASD, or who have an alcohol use disorder and do not use effective contraception [17]. This review included 50 studies; the authors rated the methodological quality of one study as ‘strong’, while 12 were rated as ‘moderate’, and 37 as ‘weak’. Of the six studies that included Indigenous populations, one was rated as ‘moderate’ and the remaining five ‘weak’.

Few prevention activities targeting Indigenous people have been evaluated. After identifying 170 FASD related prevention projects in Canada, Salmon and Clarren [18] reported that “virtually none” had been assessed, and another review identified only three published evaluations of FASD interventions [19]. Similarly, in Australia over 60 PAE and FASD prevention and health promotion resources culturally appropriate for use with Australian Aboriginal and Torres Strait Islander communities were identified [20] but few have published peer-reviewed evaluations. A systematic review of evidence-based approaches for the reduction of alcohol consumption in North American Native women who were pregnant or of reproductive age found a lack of evaluation of their effectiveness [21]. As a result, recommendations have been made for improving the quantity and quality of intervention research in this field [17, 21].

Given the higher FASD prevalence observed in some Indigenous populations, prevention in these populations is a target of Governments in Australia, Canada and the USA [22–24]. The field of FASD research has expanded greatly since the comprehensive 2011 systematic review [20], and review of prevention among Native women in the US in 2012 [24]. The current systematic review aims to provide an update on the effectiveness of interventions undertaken in Indigenous/Aboriginal populations around the World to date, and to make recommendations about effective ways forward.

## Methods

This systematic review was registered with the International prospective register of systematic reviews (PROSPERO, https://www.crd.york.ac.uk/prospero/, ID number CRD42018086212) and followed the Preferred Reporting Items for Systematic Reviews and Meta-Analyses (PRISMA) reporting guidelines (http://www.prisma-statement.org/).

### Literature search

The search strategy was based upon an earlier systematic review titled “A Systematic Review of the Effectiveness of Prevention Approaches for Fetal Alcohol Spectrum Disorder” [17]. For the current review, a comprehensive search of medical, psychological and sociological electronic databases was performed up to and including 22/08/2017. This strategy was designed by an Information Specialist and included a combination of subject heading and keyword searching [17]. In addition, reference lists of articles considered for inclusion were reviewed, and Google Scholar was searched. Only peer reviewed journal articles were considered. The list of included articles was sent to 10 experts in the field with requests for additional literature that may not have been identified by the search.

The search strategy employed by [17] was replicated for MEDLINE, PsycINFO, EMBASE, CINAHL Plus, Web of Science, and SocINDEX databases. A similar strategy was used with the Informit search engine (https://www.informit.org), which allowed for convenient searching of 15 index databases related to Aboriginal Australians. An additional pdf file shows the complete search strategies for each database (see Additional file 1). The CINAHL Plus interface was used in preference to CINAHL as it includes additional nursing and allied health literature (https://health.ebsco.com/products/cinahl-plus). The restriction of articles to Indigenous and Aboriginal people was performed after the original searches were complete and included populations referred to by authors as Indigenous and/or Aboriginal, relevant Medical Subject Headings (MeSH), and additional specific terms such as Eskimo or Maori where not already covered. A common feature of the target populations was negative impacts from colonisation. All articles were then combined into a single Endnote library and duplicates were removed.

The following steps in the review were undertaken independently by two members of the research team (MS and RP, MS and KB for data extraction), and any discrepancies were discussed with the senior author (EM) and consensus reached.

### Study selection

The titles and abstracts for articles identified by the search strategy were examined, and all articles considered relevant were downloaded for consideration for inclusion in the review. The authors of articles that could not be readily accessed were contacted and asked to provide copies. The articles were then assessed for possible inclusion against the following pre-determined selection criteria:

1. Primary research was conducted
2. The paper included any data aiming to evaluate a prevention intervention with the objective of reducing PAE or FASD
3. All types of prevention interventions were included except for training/education targeted only at the workforce
4. Data on at least one of the following outcomes was reported:
  - FASD prevalence or risk
  - Prenatal alcohol exposure
  - Knowledge, Attitudes or Intentions either around FASD or alcohol consumption during pregnancy
  - Family Planning/contraception
5. Included, and reported separately on, any Indigenous or Aboriginal population of at least adolescent age
6. In English

### Assessment of methodological quality and potential for bias

Ospina et al. [17] used a quality rating tool developed by the Effective Public Health Practice Project (EPHPP) as it could be applied to the diverse range of designs identified in their review. However, we found this tool was not appropriate for rating the quality of the studies identified in our search. Instead, the methodological quality of each study was documented and assessed using assessment tools developed by The National Heart, Lung and Blood Institute (part of the National Institutes of Health in the US) for use in conducting systematic reviews (https://www.nhlbi.nih.gov/health-topics/study-quality-assessment-tools).

### Data extraction

The following information was extracted from each paper using a pre-designed template: country where the study took place, the study population including Indigenous status and eligibility criteria, brief descriptions of the intervention and study design, the types of outcome measures used and results reported.

## Results

**Fig. 1:**
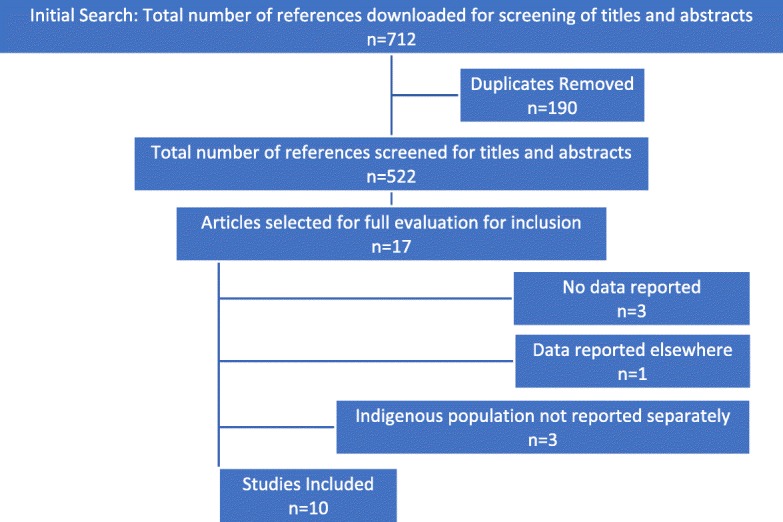
PRISMA flow chart of study selection process

The systematic search of peer-reviewed literature resulted in the identification of 712 articles: MEDLINE (98), Embase (89), CINAHL Plus (73), Web of Science (143), PsycINFO (42), SocINDEX (39) and Informit (441, 228 unique). After combining references in Endnote and removing duplicates, a total of 522 articles were identified. All papers identified in Ospina et al. [17] with Indigenous components were retrieved except for Bowerman 1997 [25] which was added manually having not been identified using their original search strategy. After initial title and abstract screening, the full text of 17 articles was reviewed and 10 met the inclusion criteria (Fig. 1). The reasons for exclusion for the remaining seven studies are shown in (Table 1).

**Table 1:** Excluded articles with exclusion criteria

Study	Reason for exclusion
Hanson et al., 2011 [69]	No data reported
Jones & Nakamura, 1993 [70]	No data reported
Ma et al., 1998 [71]	Indigenous population not reported separately, no count given
Mehl-Madrona, 2000 [72]	Indigenous population not reported separately
Montag et al., 2014 [73]	No data reported
Montag et al., 2014 [74]	Data reported elsewhere
Pascal, et al., 2014 [75]	Indigenous population not reported separately

### Characteristics of the studies included

Key characteristics of the 10 included studies are shown in Table 2. Nine of the studies were conducted in the US with American Indian/Alaskan Native communities, and one in Australia with Aboriginal people. The study populations included non-pregnant women of child-bearing age, and/or pregnant women, the general public and one study [26] included school children (Table 2). Two studies collected data from males. A range of study designs were used including one randomised controlled trial, five cohort studies with pre-post design – four of which followed-up participants at several timepoints, one cross-sectional study with different pre- and post-intervention groups, and four studies that only collected data post intervention (Table 2).

**Table 2:** General characteristics of included studies

Study	Country	Target Population	Study Population	Design	Brief Description of the Intervention	Outcome Types	Results
Indicated Strategies							
KB Masis & PA May, 1991 [29]	US, Arizona	American Indian women from Tuba City	Women referred to an Indian Medical centre at ‘high risk’ for producing alcohol affected children	Single cohort with surveys conducted post-intervention	Primary prevention included: community media, posters, and pamphlets; training of school and health personnel Secondary prevention included: screening of prenatal patients for alcohol use with education about FAS and alcohol; referral of women with high risk drinking or a child with FAS to tertiary prevention Tertiary prevention evaluated in this study: case management; counselling; detoxification; individual and group alcohol treatment, follow-up and after care; voluntary birth control or sterilisation	Number of children born with FAS-FAE Alcohol consumption: Screening tool assessment of risky drinking; self-reported frequency and quantity verified by family members at 18 months (*n* = 32) Contraceptive Use (*n* = 32)	See brief summary in text
PA May et al., 2008 [30]	US	American Indian women from four communities in Northern Plains States	Women at extremely high risk for PAE (substantial history of alcohol abuse, drinking during pregnancy or previous birth of a child with FASD)	Pre-post cohort study with data collected at baseline then at six month periods through to 72 months	Training of a prevention site manager and case manager at each of the four sites who provided three levels of FASD prevention activities. Community: education and policy strategies High risk groups: Screening, targeted messages, referral for alcohol abuse Women identified through screening: Case management enhanced by brief intervention based on MI	Number of children born with FASD Alcohol consumption: Frequency, Times “high” or drunk, binge drinking (Three drinks or more per occasion per day) Birth control status	Overall, 69.5% of the time (*n* = 105) fetuses were protected from PAE either by using birth control while drinking (39%), not drinking and using birth control (18.1%), or not drinking and not using birth control (12.4%) Further results are not reported due to high loss to follow-up from baseline (*n* = 115) to 6 months (*n* = 39) and 12 months (*n* = 37).
JD Hanson et al., 2017 [31]	US	American Indian women from two reservation sites and one urban site ≥18 years old, sexually active and fertile	Non-pregnant women All participants were at risk of AEP (4 or more drinks per occasion or 8 or more drinks per week and not using any contraception or using a method incorrectly or inconsistently)	Single cohort study with surveys at baseline, and three and six months post-intervention	Oglala Sioux Tribe (OST) CHOICES Program was delivered to all participants (2– 4 sessions) The intervention included MI techniques delivered by trained interventionists to encourage participants to decrease binge drinking and increase birth control use to reduce the risk of AEP The program supported participants to: Set goals related to alcohol use and contraception use; Complete daily diaries to track alcohol use, sexual activity and birth control use; and seek health practitioner support for birth control through referrals	Proportion of women at risk of AEP (defined as per the inclusion criteria, along with the proportion of participants pregnant at follow-up) Alcohol consumption: Volume, frequency, binge episodes Contraceptive use: Use of effective birth control and sexual activity	Significant reduction in AEP risk from baseline (100%) to three months (exact value not provided, *p* value not stated), and six months (exact value not provided, p value not stated)
Selective Strategies							
P Bridge, 2011 [27]	Australia, Ord Valley in remote North- Western Australia	Five target groups 1. All Aboriginal antenatal clients attending Ord Valley Aboriginal Health Service (OVAHS) 2. All Aboriginal women aged 13–45 years in the Ord Valley 3. All OVAHS staff 4. Local Aboriginal men 5. Broader community including national and international FASD networks	All women attending OVAHS antenatal clinics Aboriginal women aged 13–45 years in the Ord Valley	Pre-post cohort study	The following were provided to each antenatal client and extended to partners, families and the wider community: FASD education including contraception education and advice; alcohol and other drug assessment; one-to-one counselling; brief intervention; and MI Intervention quantity and consistency were not reported Community stalls and FASD workshops (33 female only, 6 male only and 23 mixed gender) OVAHS staff received FASD education including contraception education and advice and training in brief interventions for alcohol and contraception use and MI	Antenatal clients completed routine assessments at three times during pregnancy Alcohol consumption Proportion of unplanned pregnancies Proportion of people receiving FASD education, satisfaction with education received	See brief summary in text
JD Hanson et al., 2013 [28]	US, Northern Plains	American Indian women from three tribes	Non-pregnant, sexually active women who had consumed alcohol in the past three months	Descriptive longitudinal cohort study with surveys at baseline and every three months for one year	Brief interventions based on MI delivered by phone with supporting intervention materials mailed to participants	Risk of AEP (> 4 drinks in a day or > 7 drinks in a week or no protection at any one point or failure to not always use a contraceptive method or both) Alcohol consumption in the past 90 days: Most drinks at any one time, average drinks per day, average drinks per week, and how many times consumed 3+ drinks Birth control use (*n* = 162) in past 90 days: sexual activity, contraception method, frequency	All categories of alcohol consumption showed decreases over time Birth control use increased at three months and remained consistent across the rest of the study Due to loss to follow-up detailed results are not reported
AC Montag et al., 2015 [34]	US, Southern California	American Indian/Alaskan Native women	Women of childbearing age recruited at three AIAN health clinics	Randomised control trial of an intervention compared with treatment as usual with surveys at baseline and one, three and six months post-intervention	~ 20-min web-based brief assessment and intervention tool tailored to the population consisting of an anonymous survey followed by individualised risk feedback for AEP, including impact of alcohol exposure on a fetus, physical and financial costs of alcohol consumption and comparison of drinking levels with other Native women Information on additional resources was provided at the end of the web session and could be printed Treatment as usual control: Assessment of alcohol use and access to displayed health education brochures that did not include FASD specific information	Proportion of women at high risk of AEP Alcohol Consumption: number of drinks per week and per occasion, number of binge episodes (> = 3 drinks) in past 2 weeks. Birth control use (only reported at baseline). Awareness of FASD and knowledge regarding the risks of alcohol consumption for women and their pregnancy (baseline only)	All outcomes showed a significant time effect but no intervention effect. The proportion of women at high risk of AEP (%) for the intervention and control groups respectively was 36.4/33.6% at baseline, 18.8/21.9% at one month, 16.7/21.7% at three months and 18.9/22.1% at six months. Drinks per week were 4.40 ± 0.94, 0.89 ± 0.21, 0.98 ± 0.26, and 1.64 ± 0.55 for the intervention group and 3.38 ± 0.50, 1.34 ± 0.24, 1.94 ± 0.38, and 1.99 ± 0.46 for the control group at baseline, one month, three months and six months respectively Binge episodes (over two weeks) were 1.47 ± 0.40, 0.36 ± 0.08, 0.49 ± 0.17, 0.50 ± 0.12 for the intervention group and 1.06 ± 0.16, 0.49 ± 0.09, 0.62 ± 0.13, 0.72 ± 0.14 for the control group at baseline, one month, three months and six months respectively
Universal Strategies							
PA May & KJ Hymbaugh, 1989[26]	US-wide	Native American and Alaskan Native communities serviced by 92 Indian Health Services across 48 USA states	Prenatal groups, school children, Indian Health Service (IHS) workers and community groups	Pre-post intervention surveys with multiple disparate cohorts and limited follow-up	The National Indian FAS Prevention Program was developed to provide knowledge, skills and educational resources for communities to carry out primary through tertiary prevention Resources and materials developed for FAS prevention were keyed to one of the four target groups. Resources included ten pamphlets, six posters, 16 fact sheets and a set of 20 slides with accompanying narrative from a trained educator Provision of training to Indian Health Service FAS trainers and personnel included a two-day workshop on clinical and educational interventions. Further phone and correspondence monitoring and coaching of trainees was also provided.	Five questionnaires were used to assess prevention education. Four consisted of fact identification and fixed response items and one had eleven open ended questions for adults. FAS Knowledge	Four of eight school classes (from Grade 5 through to high school) had significant improvements (*p* < .05) in knowledge pre- to post-test Eleven of 14 adult groups had significant improvements in knowledge (*p* < .01) pre- to post-test Two out of three groups (two high school and one community health group) had significant knowledge gain (*p* < .01) from pre- to post-test after receiving the education materials alone Of the six groups assessed at follow-up at least two months later (four school classes and two community groups), four had significant (*p* < .01) knowledge gain post-education and three were still significantly (*p* < .01) higher at follow-up compared with pre-test
KJ Plaisier, 1989 [33]	US, Michigan’s Upper Peninsula	American Indian Communities	Women of childbearing age who were pregnant or had delivered an infant in last 12 months were recruited at clinics or by Indian health workers	Cohort intervention with post intervention survey	Indian health workers were educated using previously developed culturally sensitive materials, and helped to plan and deliver FAS education programs. Programs aimed to encourage women to participate in sponsored community-wide workshops, including school and senior citizen programs. Individual counselling was provided at clinics	FAS Knowledge	See brief summary in text
RJ Bowerman, 1997 [25]	US, Alaska	American Indian and Alaskan Native populations	Pregnant women from six remote villages in Barrow in Arctic Alaska	Cross-sectional pre- and post-intervention surveys with different groups	1994 ban on alcohol possession in the town of Barrow	Alcohol consumption: reported by trimester as percentage engaged in “alcohol abuse” (not defined)	The proportion of women engaged in alcohol abuse was reported as 42% for the pre-intervention sample and 9% for the post-intervention sample (RR = 0.21, 95% CI = 0.08, 0.55) Alcohol abuse in the 1st Trimester was reported as 43% pre-intervention and 11% post-intervention (RR = 0.25, 95% CI = 0.07, 0.94) Differences in pre- and post-intervention groups for Trimester 2 (17% and 7%) and Trimester 3 (14% and 5%) were not significant (RR, p value, and CI not reported)
JD Hanson et al., 2012 [32]	US	Three tribal American Indian communities located 400–600 miles apart in the Northern Plains	American Indian Women of child bearing age (18–44 years) self-enrolled by calling a 1–800 phone line	Post-intervention evaluation	A culturally and linguistically tailored media campaign included: posters displayed in community settings and local newspapers; radio advertisements; pens; brochures; and t-shirts. The campaign was delivered through: information booths set up at local fairs or community events, community centres, health clinics and local tribal colleges; community presentations at local schools and treatment facilities; and Public Service Announcements and live interviews broadcast on local radio stations	Post-campaign telephone surveys assessed participants attitudes regarding the effects of the campaign including: Alcohol consumption FASD knowledge Cultural appropriateness of the campaign	See brief summary in text

### Types of intervention

Four of the 10 studies employed *universal* level interventions which included school-based, community-wide and one-on-one education campaigns, and a community-wide alcohol ban. Four [27–30] of six studies evaluating *selective* (*n* = 3) or *indicated* (*n* = 3) strategies were conducted in the wider context of concurrent *universal* prevention activities. All *selective* and *indicated* strategies used case-management and/or screening for alcohol use with brief intervention and/or referral to specialised treatment. In addition, three of these studies [28, 30, 31] included principles of motivational interviewing (MI). Interventions were delivered either face-to-face, by telephone or via the internet. The intensity of delivery varied from a single 20-min session to an average of 19 case management contacts per participant. Non-pregnant women were recruited in three studies, pregnant women in two and one study recruited both. In two studies the women were described as ‘at high risk of an alcohol exposed pregnancy (AEP)’, ‘at risk of an AEP’ in two more studies, with the remaining two offering the intervention to all women.

Except for the community-wide alcohol ban in [25], all studies described their interventions as culturally appropriate or modified for use with local populations, typically in collaboration with local Indigenous community members. Five studies [26, 27, 29, 32, 33] explicitly reported that local Indigenous staff implemented the interventions, while this was not made clear in the remaining studies.

### Outcomes assessed

Of the seven studies in which alcohol consumption was an outcome measure, all used self-reported assessment; intake was classified variously as: average drinks per week, average or maximum drinks per drinking occasion, and frequency of binge drinking (defined as ≥3 or ≥ 4 standard drinks per occasion) with a US standard drink containing roughly 14 g of pure alcohol. The Alcohol Use Disorders Identification Test was explicitly identified as the measurement tool in one study [30] and implicitly in another two studies [28, 31]. A 10-item questionnaire from Boston hospital was used in one study [29], and the remaining three studies [25, 27, 34] used their own set of self-report questions with no reported validation. The use of family planning/birth control/contraception was also assessed. Specifically, four of the 10 studies [28, 30, 31, 34] combined the measurement of alcohol and contraception to assess the ‘risk of an alcohol exposed pregnancy’. Two studies [29, 30] reported the number of children born with FASD during the study period. Studies employing *universal* strategies assessed reported alcohol use in pregnant women, knowledge of FAS or the risks associated with alcohol consumption during pregnancy in individual women, in school classes, community and staff groups. One of these studies assessed knowledge of FASD rather than FAS. The outcomes assessed for each study are shown in Table 2.

### Summaries of included papers

Brief summaries of the 10 included papers, organised by level of intervention and year, are provided below with additional details of the study characteristics and results available in Table 2.

#### Indicated approaches

##### Masis & May, 1991 [29]

Women classified as “high risk” of an AEP were referred for case management (48 recruited, 39 participated) to address their alcohol consumption in the context of a FAS prevention program using primary, secondary and tertiary methods. The interpretation of study results was hampered by inconsistent reporting. At assessment after 18-months (*n* = 32), the proportion of women abstaining from alcohol was reported as 56.3% with 12.5% reporting drinking less. The grouping and reporting of pregnant and non-pregnant participants was difficult to follow and it was not clear which of these women were pregnant. Women referred during pregnancy were reported on separately; 21 of the 29 referred participated in the program. In total 18 (85.7%) of these women reported abstaining from alcohol during pregnancy although it was not clear when this information was collected and could represent abstinence achieved at the third trimester. After 18 months, 10 women reported using birth control, six had voluntary tubal ligation and eight had become pregnant. The denominator for percentages reported for birth control use included women lost to follow-up. It was not possible to determine how many pregnancies were at risk of alcohol exposure due to the separate reporting of alcohol use and contraception. Of 11 FAS-FAE (Fetal Alcohol Effect) births reported, eight were born to participants before the study, two were born to women who had not yet been recruited and one child was born to a woman recruited to the study who had refused services. Overall, it was unclear if AEP risk was reduced as a result of the intervention, and the considerable non-participation (19%) and loss to follow-up (18%), small sample size, and lack of baseline measurements rendered the results of this study susceptible to bias; therefore, the methodological quality was rated as ‘Poor’.

##### May et al., 2008 [30]

This study, conducted in four North American Indian communities, recruited women at high risk of drinking during pregnancy to undertake MI enhanced case-management to address their alcohol consumption and general wellbeing in the context of a simultaneous community education program. Participants were enrolled in the program for one to 98 months (mean 17 months) with an average of 19 contacts with intervention staff. At enrolment, participants completed multiple assessments designed to ascertain the prior prevalence of FASD in the communities and provide personalised risk feedback to increase motivation for change. Shorter batteries of instruments were given every 6 months until 72 months had passed. However, alcohol use was only reported at baseline and at 6- and 12-months post-intervention. At baseline, 67.9% of women reported abstinence over the previous 6 months. The study reported that many clients had commenced the program after a situation encouraging abstinence (e.g. incarceration). Reported abstinence was lower at 6 months and significantly lower at 12 months. While there was a significant reduction in the proportion of pregnant women who reported being drunk three or more times in the previous 6 months, there was a concurrent increase in the proportion of women who reported drinking three or more drinks on a typical drinking day in the previous 30 days. Reported contraceptive use increased between baseline and 12 months. Using data from 6-, 12-, 18- and 24-month follow-ups, the authors stated that for 69.5% of the time, non-pregnant women were “protected” against AEP as they were either not drinking, using birth control, or both. Two of the 119 children delivered by participants were born with suspected FASD. At baseline, 23.8% (*n* = 41) of the 172 women who completed screening declined to take part. Of the 137 women who participated, loss to follow-up at six months (66.1%) and 12 months (67.8%) was high. Thus, while the authors reported a reduction in risk of AEP following the intervention, the methodological quality of this study was rated as ‘Poor’.

##### Hanson et al., 2017 [31]

A pre-existing intervention based on MI – known as Project CHOICES (Changing High-Risk AlcOhol Use and Increasing Contraceptive Effectiveness Study) – was modified for use with three Oglala Sioux tribes in the US. Multiple strategies were used to recruit 193 non-pregnant women at risk of AEP. Surveys were conducted with women at baseline and again at 3- and 6-month follow-ups after attendance at two to four intervention sessions. A significant decrease in the proportion of pregnancies at risk for AEP was found between baseline (100%) and 3 months (25–47%) and baseline and 6 months (18–66%). These percentage ranges reflect assumptions that all drop-outs were either ‘not at risk’ or ‘at risk’ (respectively) at follow-up. Even when all drop-outs were considered to have been at risk, there was still a significant reduction of AEP risk over time. From 102 responses available at 3 months, risk reduction was mostly achieved by the use of birth control (67.7%), rather than reducing binge drinking (9.8%) or using both methods (22.6%). Although follow-up was reported as successful (78.8% of women completed one follow-up and 51.3% completed both) these figures, together with the lack of a control group, limited confidence in the overall findings. Therefore, the study methodology was rated as ‘Poor’.

#### Selective strategies

##### Bridge, 2011 [27]

In the only study conducted outside North America, Australian Aboriginal women (*N* = 78) living in remote Northern Australia were provided with FASD and contraception education, alcohol and drug assessment and brief intervention, and MI where applicable, in the context of a broad-based FASD prevention strategy. Routine assessments were conducted three times during pregnancy through attendance at antenatal clinics, although only four of the 78 participants completed all three assessments. Most of the women (84.5%) consumed alcohol at some time during the pregnancy, often before pregnancy recognition. After receiving FASD education, 56.4% of all participants reported abstaining from alcohol for the rest of pregnancy, 14.1% reduced alcohol consumption, and 1.2% continued to drink alcohol (level not stated). Two participants refused FASD education and 10.2% were lost to follow up because they had moved to another location. The majority of pregnancies were unplanned (70.5%). It was also reported that FASD brief interventions were provided to over 50% of 770 local Aboriginal women and some men through community events and education workshops. Participants considered the interventions very useful for themselves and their families; however, a subsequent reduction of risk of PAE was not clearly demonstrated. The low proportion of women who completed all three assessments, missing data pertaining to the assessment of FASD knowledge, and lack of methodological detail contributed to a ‘Poor’ methodology rating.

##### Hanson et al., 2013 [28]

This study was the *selective* arm of a wider *universal* prevention strategy described in [32]. Non-pregnant American Indian women who were sexually active and consumed alcohol in the past 3 months self-enrolled via an advertised phone hotline. Brief interventions based on MI constructs were provided by phone with additional intervention materials adapted from Project CHOICES mailed to participants at each follow-up. Surveys about alcohol consumption and the use of birth control were conducted at baseline (*n* = 213 and 162 respectively), and at 3- (*n* = 120 and 78), 6- (*n* = 81 and 52), 9- (*n* = 62 and 34) and 12-month follow-ups (*n* = 51 and 30). The proportion of women deemed to be at risk of an AEP was 54% at baseline and 20% at 12 months, although these figures were inconsistent with the reported use of birth control. The proportion of women lost to follow up was 43.7% at 3 months increasing to 76.1% at 12 months. Therefore, the potential for selection bias was high and the results are unlikely to be valid. The recruitment by self-selection may have introduced bias and it is unclear how the concurrent universal prevention strategy may have influenced the effectiveness of the intervention. Given these factors, the methodological quality of this study was rated as ‘Poor’.

##### Montag et al., 2015 [34]

This was the only randomised controlled trial identified in this systematic review. American Indian/Alaskan Native (AIAN) women of childbearing age (*n* = 263) were recruited at health clinics and randomised to either a culturally targeted 20-min online alcohol screening and brief intervention or to receive treatment as usual with access to standard clinic brochures with no FASD specific information. The main outcome assessed was the proportion of women at high risk for an AEP. This was defined as drinking three or more standard drinks per occasion or eight or more drinks per week, and using a contraceptive method considered by WHO guidelines to be less than highly effective. In the intervention group (*n* = 113), the proportion of women classified as high risk reduced from 36.4% at baseline, to 18.9% at 6 months. In the control group (*n* = 134), these figures were 33.6% to 22.1%. Thus, while both groups showed an apparent decline in risk of AEP over time, there was no significant difference between the intervention and control groups. Methodological issues identified by the authors included flaws in the randomization procedure, reliance on self-report which may have been influenced by social desirability response biases, and a mixed-mode data collection process where the intervention group completed web-based questionnaires and the control group by telephone. The blinding process for participants could not be determined. All participants had multiple opportunities to request referral to treatment with a professional substance abuse counsellor although the number of women who received this additional treatment was not reported. Women in the control group may have been more inclined to seek referral, not having received the brief intervention. If this occurred, it may have masked the effectiveness of the intervention and biased the results to the null. Due to the combination of issues the methodological quality of this study was rated as ‘Poor’.

#### Universal

##### May & Hymbaugh, 1989 [26]

This study was conducted in the context of a multi-level FAS prevention program that included training of advocates in local Indian Health Services in primary, secondary and tertiary prevention techniques, and the development of resources targeted to specific subsets of the local community. The trained workers then provided education about FAS to school children, community members, pregnant women and health workers using a set of 20 slides accompanied by narrative scripts. To assess changes in knowledge in these groups, surveys were conducted pre- and post-intervention with additional follow-up after 2 months for six groups. Five different surveys were used in the study making comparison between groups difficult. There were significant knowledge gains in four of eight school classes and in 11 of 14 adult groups. Three of six groups tested at follow-up showed significant knowledge retention, although loss to follow-up was greater than 20% in four of the groups. Two of the three groups exposed to the materials alone without the trainer narratives showed significant knowledge gain. The sample size for some of the individual groups was small with 21 of the 31 samples having 20 or fewer participants. In addition, because of the use of multiple different questionnaires, lack of control group, and the inability to determine if all eligible participants were enrolled, the methodological quality of this study was rated as ‘Poor’.

##### Plaisier, 1989 [33]

Education about FAS was provided to communities by American Indian health workers trained to deliver community workshops, school and senior citizen programs, parental education packages and one on one counselling. The educational materials used were sourced from two pre-existing packages, one of which was described as culturally sensitive. American Indian women from the communities, who were pregnant or had delivered a child within the last year, participated in interviews that assessed their knowledge of FAS. These assessments were only conducted post-intervention. Of the 29 women who took part, 83% said that they had heard of FAS, 50% could describe at least one specific characteristic of FAS, and 25% said that no amount of alcohol was safe to drink during pregnancy whereas 75% didn’t know the safe consumption level. Only 10% had learned about FAS from their physician and 39% recalled their physician discussing alcohol and pregnancy with them. Several issues that contributed to the ‘Poor’ methodological quality rating of this study included small sample size, lack of detail about the survey, lack of information about the extent of exposure to the intervention, and no baseline assessment of knowledge. Further, the authors pointed out that women recruited to the study were well connected to the health service. Those who were more socially isolated, and potentially more likely to be at risk of alcohol problems, were not included.

##### Bowerman, 1997 [25]

This letter to the editor described a community supported alcohol possession ban in a small community in Alaska with a high proportion of Alaskan Natives. It was reported that all known pregnant women in the community were provided with standard prenatal care and FAS education. Alcohol consumption self-reported by 275 pregnant women before the ban was compared with that of 73 women after the ban. The proportions considered to have been abusing alcohol were 42% and 9% respectively. The biggest difference pre- and post-ban was observed for the first trimester of pregnancy. The tool used to assess alcohol consumption and the definition of alcohol abuse were not described. Additionally, the lack of methodological detail and the absence of a control community contributed to a ‘Poor’ methodology rating.

##### Hanson et al., 2012 [32]

This study aimed to evaluate a culturally and linguistically tailored media campaign focused on FASD awareness and prevention. A wide range of resources were developed for the campaign and delivered using grassroots outreach activities. A convenience sample of 119 American Indian women of childbearing age who had self-enrolled in a concurrent FASD prevention project [28] responded to a survey about their opinions of the media campaign. Most agreed or strongly agreed that the campaign was culturally appropriate (86%) and that the campaign had increased their knowledge of FAS (92%) and the effect of alcohol on the unborn child (93%). Almost three quarters (72%) also agreed/strongly agreed that the campaign had decreased their alcohol consumption. However, no baseline data were collected, and the survey relied on the use of agreement scales which were unable to directly assess the campaign’s effect on knowledge or behaviour. Consequently, the methodological quality was rated as ‘Poor’.

In summary, the collective evidence regarding the effectiveness of FASD prevention approaches in Indigenous communities was weak with the methodological quality of all 10 studies rated as ‘Poor’. While two studies reported improvements in FASD knowledge, and one study reported a reasonable level of knowledge had been achieved, and a further five reported a reduction in risk of PAE mostly achieved through the increased use of birth control rather than decreased drinking, there were major flaws in study design. The main issue was the lack of control group for all studies apart from Montag et al. [34]. Substantial loss to follow-up was a problem common to many of the studies. It was often difficult to determine the proportion of women who declined the invitation to participate in the intervention. Both alcohol consumption and use of contraception were self-reported, and the validity of the instruments used for this purpose was not reported in many of the studies. For the six studies that assessed individual interventions, four were performed in the context of wider ranging *universal* prevention efforts which may have introduced a potential source of bias to the study results.

## Discussion

Ten studies conducted over a period of 28 years from 1989 met the inclusion criteria for this systematic review. The study designs, target populations, and interventions delivered in these studies were heterogeneous making comparison of effects difficult. Overall, there is little evidence that any of the interventions were effective in reducing the risk of PAE or FASD in the study populations. The results of all studies were subject to substantial biases because of high loss to follow-up, lack of a control group and the reliance on self-report measures to assess the main outcomes. The only randomized controlled trial reported no difference in outcomes between the intervention and control groups. The same limitations were found in a systematic review of prevention of PAE in the general population [17], and two previous systematic reviews of approaches to reduce alcohol consumption in American Indians/Alaskan Natives [21] and Australian Aboriginal settings [35].

Education and media campaigns aimed at increasing knowledge of FASD and the harmful effects of alcohol consumption during pregnancy were the target of three studies in this review. However, only one study assessed changes in knowledge using both pre- and post-intervention assessments. While addressing gaps in knowledge may be a necessary component of prevention efforts, health promotion and behaviour change frameworks coupled with evidence from the smoking and alcohol fields suggest that the provision of education alone is not sufficient to change behaviour [36–38]. This may be especially true for individuals at greatest risk or disadvantage [39, 40]. Hence in addition to examining gains in knowledge, future work should seek to determine whether such gains lead to improvements in alcohol consumption or contraceptive use in the target population.

The interventions aimed at reducing alcohol intake and increasing use of birth control were largely focussed on changing the behaviour of women at risk of PAE, and were typically more intensive. All but one study [29] included both pre- and post-intervention assessments, often with multiple post-intervention follow-ups. There was generally greater emphasis placed on the assessment and prevention of alcohol consumption than addressing birth control and, importantly, none of the studies targeted birth control in isolation. Only one study [34] considered the effectiveness of specific contraceptive methods in assessing PAE risk, and future work integrating this approach would be valuable. Findings from studies using both approaches suggested that women may be more likely to change their contraceptive behaviours than reduce their alcohol consumption. Further exploration of the interaction between these two prevention pathways would be informative.

All studies assessed alcohol consumption using self-report measures. Obtaining accurate self-report data on alcohol consumption during pregnancy can be affected by factors such as stigma, recall error, confusion regarding the definitions of levels and patterns of intake, and researchers not following best practice during administration. While all relevant studies reported staff training in intervention delivery, little or no information was provided regarding training for assessing alcohol exposure and contraception. Surveys were conducted by phone, online, or in the presence of a researcher. If this resulted in under-reporting of alcohol consumption because of social desirability [41] the effectiveness of some interventions may be over-estimated. One study [27] reported following recommendations to elicit more accurate self-reported alcohol consumption data from pregnant women, such as ensuring that questions are asked in a non-judgemental manner, as part of normal practice for everyone, in a welcoming environment [41].

Many commonly used instruments, such as the AUDIT-C, were designed to detect ‘at risk’ drinking or alcohol abuse rather than quantity of consumption during pregnancy [42]. Self-reported alcohol use during pregnancy has been found to be inaccurate when compared with objective measures such as biomarkers [43, 44]. Thus, the use of biomarkers of alcohol consumption alongside self-report has been recommended [45] although these also need further research to increase accuracy [46]. Where this is not possible, validated instruments that assess the frequency and quantity of alcohol consumption and binge drinking should be administered by trained research staff, and using lists of drink choices and measures of occasional drinking may provide more accurate measures of dose [41].

Inconsistencies in recording of alcohol consumption can be exacerbated in Indigenous populations due to language, contextual and cultural differences [47]. Ideally measures should be piloted, adapted, and validated in the specific study population to ensure cultural appropriateness. Although widely recommended, this is expensive and may not be the most cost-effective use of scarce health resources. Potential alternatives include: validating a smaller set of key questions, employing local community navigators to assist with surveying and developing local pictorial aids [48]; detailed consultation with community representatives when developing or adapting surveys [47]; or using more visual Aboriginal-specific computer applications that assist in accurate estimation of consumption, and incorporate sharing among groups and episodic patterns of drinking [49]. Another framework recommends balancing community-tailored and common approaches, the latter constrains costs and enables comparisons with external populations [50].

No study examined outcomes beyond a 6- or 12-month follow-up period. This is a relatively short time-frame given that patterns of alcohol consumption and contraceptive use may change over time and alcohol dependence can be a chronic, recurring problem. Therefore, it is likely that interventions of greater intensity and duration are necessary [51].

In a secondary analysis, one study investigated possible effects of psychosocial variables on the effectiveness of the intervention [34]. Specifically, the reduction in drinks per week reported at follow-up was influenced by engaging in binge drinking, needing treatment for depression or feeling functionally impaired, and thinking other women drink more than they do. The consideration of similar psychosocial variables in the design of future interventions will be important in elucidating factors that may influence their effectiveness.

Additionally, understanding the social determinants of women’s alcohol consumption during pregnancy will be essential for designing and evaluating the effectiveness of future interventions. Systemic factors such as access to treatment services, the effects of colonisation, loss of land and culture and economic exclusion have impacted on the way in which alcohol is consumed in Indigenous communities in Australia [52] and around the world [53]. Many complex individual factors contribute to increased risk of PAE including addiction, poor mental health, isolation, stress, low self-esteem, adverse early life experiences, racism, pre-pregnancy alcohol consumption, and exposure to violence or abuse [27, 54, 55]. Finally, societal factors such as living with a partner who consumes alcohol during the pregnancy [56], lack of social support, social motives for drinking [57], and social exclusion [55] also contribute.

One objective of studies in this review was to reduce FASD prevalence. However, only two studies reported the number of children born with FASD, and the absence of a control group made the significance of such figures difficult to determine. Future studies aiming to reduce rates of FASD should evaluate the impact of prevention efforts on FASD prevalence, although doing so certainly presents challenges. FASD prevalence is expensive and time consuming to ascertain. It is also often diagnosed later in life, particularly during school years when learning and behavioural problems become more apparent. This is especially the case for individuals without the characteristic facial features and those with milder impairments [58]. Longer term follow-up would provide more accurate estimates of FASD prevalence.

Strengths of this systematic review included adherence to PRISMA guidelines, use of a predefined search strategy, and having two reviewers independently undertake study selection and data extraction with the oversight of a senior colleague. A potential limitation was the exclusion of the ‘grey literature’. Government organisations and community groups involved with FASD prevention who have not published their research in journals would have therefore been overlooked. The decision to include only peer-reviewed papers was expected to provide a higher level of confidence in the quality of the evidence identified. Studies in specific Aboriginal or Indigenous populations not identified by either of those terms may not have been identified by the database search.

Undertaking FASD prevention research in Indigenous populations is challenging. Most studies identified in this review were conducted in regional and remote areas which can present logistic and financial barriers [59]. High loss to follow-up was also a significant problem even though studies typically attempted to mitigate this issue by using multiple contacts for participants, and providing incentives and additional support for continued attendance. Retaining participants in studies of severe alcohol use disorder is difficult and can be more challenging than for other addictive substances [60]. In health care research, the number of strategies used was found to be positively correlated with participant retention [61]. In alcohol research specifically, better retention was achieved by making more attempts to communicate with non-responders, using modern technologies to contact participants, and encouraging positive alliance and study ownership among participants [62]. Involving local community leaders to develop strategies that incorporate these approaches is an important area of research.

Prevention efforts can be hampered by language barriers and cultural differences [59]*.* To ensure culturally appropriate, reliable and sustainable interventions for Indigenous peoples, it has been recommended to include members from the priority population in all steps of program planning, integrate culture into the interventions when creating or adapting program components [63, 64], and conduct evaluations with the support and inclusion of communities [65]. Despite being detrimentally impacted by similar consequences of colonisation, Indigenous populations around the World are heterogenous. Therefore, when using existing interventions or methods in a new population, formative work is imperative even though it may be expensive and time consuming.

Despite the limitations discussed above, the studies included in this review had some strengths. Nearly all studies explicitly included local Indigenous people in the research design and methodology addressing issues such as community involvement and cultural appropriateness. Five studies also explicitly mentioned capacity building in the training and/or employment of local Indigenous staff for either implementation of the programs or data collection. Five studies had repeated measurement of outcomes, with at least one follow-up more than 2 months after the post-intervention data collection, and four of these studies had at least two follow-ups. The wide scope of the programs in targeting different population groups acknowledges the important fact that there are multiple avenues and times during the life-course that might be appropriate for addressing prevention. The multi-pronged approaches of studies offering different components targeting knowledge (FASD education), behaviour change (eg. MI or SBIRT) and practical components (eg. alcohol detoxification, provision of birth control) are logically appealing and the contribution of each strategy might be teased apart using carefully designed methodologies. Similarly, further study of approaches combining strategies at both the individual and community levels is important. Future work could incorporate some, or all, of these elements in addressing the behavioural and environmental factors that lead to PAE, whilst ensuring that each component is founded on sound theoretical models of health behaviour [66, 67]. Lessons can be learnt from smoking intervention research where the use of such models has provided direction for successful program design and consequent behaviour change [67, 68].

## Conclusion

FASD is a preventable condition which has a detrimental impact in some Indigenous communities around the world. It leads to lifelong physical, behavioural, and cognitive impairments, and the emotional and financial burden to families and community is substantial. It is concerning that none of the studies identified in this review adequately evaluated prevention efforts. FASD prevention in Indigenous communities could benefit from systematic approaches to program design, implementation, and evaluation [67]. High quality intervention trials with rigorous evaluation methods need to be conducted before any conclusions can be drawn about how best to prevent FASD. Future work should aim to include a control group/community, randomize the assignment of the intervention wherever possible, seek guidance from community members regarding ways to maximise follow-up, include objective outcome measures, and report results clearly and accurately. Funding for such work must take into account the costs involved in engaging community members in the development and conduct of the research, creating, adapting and testing resources and interventions, working in remote areas and employing specialised research staff.

## Additional file

Additional file 1: There is one additional file available in Adobe PDF format (Additional File 1.pdf) which has a table of the complete search strategies used for each database. (PDF 33 kb)

## Abbreviations

AEP: Alcohol Exposed Pregnancy (This abbreviation has only used where it has been used in individual studies, otherwise PAE has been preferred)
FAS: Fetal Alcohol Syndrome
FASD: Fetal Alcohol Spectrum Disorder
MI: Motivational Interviewing
PAE: Prenatal Alcohol Exposure
SBIRT: Screening, Brief Intervention, and Referral to Treatment
