# Supplementary material for: A systematic review of prevention interventions to reduce prenatal alcohol exposure and fetal alcohol spectrum disorder in indigenous communities
Source: BMC Public Health. 2018 Nov 3;18:1227. doi: 10.1186/s12889-018-6139-5 (PMC6215602; doi:10.1186/s12889-018-6139-5)
Supplement: Supplementary file 1 — There is one additional file available in Adobe PDF format (Additional File 1.pdf) which has a table of the complete search strategies used for each database. (PDF 33 kb) [file 12889_2018_6139_MOESM1_ESM.pdf]

Additional File 1: Complete Search Strategies for each Database

Database	Search Strategy
Cochrane Database of Systematic Review	f?etal alcohol in Title, Abstract or Keywords or (alcohol * or ethanol) AND (pregnan * OR fetus OR fetal OR prenatal OR in utero OR intrauterine) in Title, Abstract or Keywords
MEDLINE (OVID Interface) includes in process	1. Fetal Alcohol Spectrum Disorders/<br>2. f?etal alcohol.tw.<br>3. ((alcohol* or ethanol) adj3 (birth defects or congenital malformations or neurodevelopmental)).mp.<br>4. fasd.tw.<br>5. fae.tw.<br>6. arnd.tw.<br>7. arbd.tw.<br>8. 1 or 2 or 3 or 4 or 5 or 6 or 7<br>9. Alcoholism/<br>10. Alcoholic Intoxication/<br>11. Alcohol-Induced Disorders/<br>12. Alcohol-Related Disorders/<br>13. Substance-Related Disorders/ and (alcohol* or ethanol).tw.<br>14. exp Alcoholic Beverages/<br>15. Alcohol Drinking/<br>16. Ethanol/<br>17. 9 or 10 or 11 or 12 or 13 or 14 or 15 or 16<br>18. fetus/<br>19. pregnancy/<br>20. pregnancy, high-risk/<br>21. exp pregnancy outcome/<br>22. prenatal injuries/<br>23. prenatal exposure delayed effects/<br>24. pregnancy complications/<br>25. maternal exposure/<br>26. 18 or 19 or 20 or 21 or 22 or 23 or 24 or 25<br>27. 17 and 26<br><br>The following two searches were not used:<br>((alcohol * or ethanol or (drinking not drinking water)) and (pregnan * or fetus or fetal or prenatal or in utero or intrauterine)).tw.<br>limit 28 to (“ in data review ” or in process or “ pubmed not medline ”)<br><br>28. 8 or 27<br>29. Fetal Alcohol Syndrome/pc<br>30. Preventive Health Services/<br>31. primary prevention/<br>32. secondary prevention/<br>33. tertiary prevention/<br>34. public policy/<br>35. health policy/<br>36. house calls/<br>37. risk reduction behavior/<br>38. prevent*.tw.<br>39. ((reduc* or lower or decrease* or smaller) adj3 risk*).tw.<br>40. protect*.tw.<br>41. best practice*.tw.<br>42. exp Communications Media/<br>43. Social Marketing/<br>44. Product Labeling/<br>45. campaign$.mp.

	46. counseling/<br>47. government programs/<br>48. Community Health Services/<br>49. health promotion/<br>50. exp Health education/<br>51. Social Control Policies/<br>52. Prenatal Care/ and ((intervention* or trial).ti. or ((reduc* or lower) adj3 (alcohol or drinking or substance)).tw.)<br>53. program evaluation/<br>54. 30 or 31 or 32 or 33 or 34 or 35 or 36 or 37 or 38 or 39 or 40 or 41 or 42 or 43 or 44 or 45 or 46 or 47 or 48 or 49 or 50 or 51 or 52 or 53<br>55. (28 and 54) or 29<br>56. limit 55 to animals<br>57. 55 not 56<br>58. limit 57 to english language<br>59. exp Oceanic Ancestry Group/<br>60. exp American Native Continental Ancestry Group/<br>61. Population Groups/<br>62. (Aborig* or Indig*).mp. [mp=title, abstract, original title, name of substance word, subject heading word, floating sub-heading word, keyword heading word, protocol supplementary concept word, rare disease supplementary concept word, unique identifier, synonyms]<br>63. 59 or 60 or 61 or 62<br>64. 58 and 63
Embase (OVID Interface)	1. exp Fetal Alcohol Syndrome/<br>2. fetal alcohol.tw.<br>3. (alcohol* adj3 (birth defects or congenital malformations or neurodevelopmental)).tw.<br>4. (alcohol* adj4 prenatal).tw.<br>5. (fasd or fae or arbd or arnd).tw.<br>6. 1 or 2 or 3 or 4 or 5<br>7. alcohol/<br>8. alcoholism/<br>9. alcohol abuse/<br>10. alcohol drinking patterns/<br>11. alcohol intoxication/<br>12. exp alcoholic beverages/<br>13. alcohol consumption/<br>14. drinking patterns/<br>15. 7 or 8 or 9 or 10 or 11 or 12 or 13 or 14<br>16. pregnancy/<br>17. fetus/<br>18. prenatal exposure/<br>19. 16 or 17 or 18<br>20. 15 and 19<br>21. 6 or 20<br>22. Fetal Alcohol Syndrome/pc<br>23. "prevention and control"/<br>24. protection/<br>25. primary prevention/<br>26. secondary prevention/<br>27. tertiary prevention.mp.<br>28. health education/<br>29. prevention/<br>30. health promotion/<br>31. policy/<br>32. social marketing/<br>33. health care policy/<br>34. health program/<br>35. harm reduction/

	36. risk reduction/<br>37. best practice*.tw.<br>38. ((reduc* or lower or decrease* or smaller) adj3 risk*).tw.<br>39. prenatal care/<br>40. 23 or 24 or 25 or 26 or 27 or 28 or 29 or 30 or 31 or 32 or 33 or 34 or 35 or 36 or 37 or 38 or 39<br>41. (21 and 40) or 22<br>42. limit 41 to english language<br>43. (exp vertebrate/ or animal/ or experimental animal/ or nonhuman/ or animal.hw.) not exp human/<br>44. 42 not 43<br>45. exp Indigenous people/<br>46. exp Oceanic ancestry group/<br>47. exp American Indian/<br>48. exp eskimo/<br>49. exp "Maori (people)"/<br>50. (Aborig* or Indig*).mp. [mp=title, abstract, heading word, drug trade name, original title, device manufacturer, drug manufacturer, device trade name, keyword, floating subheading word]<br>51. 45 or 46 or 47 or 48 or 49 or 50<br>52. 44 and 51
CINAHL Plus (Ebsco Interface)	S1: fetal alcohol<br>S2: ((MH " Alcoholic Intoxication ") or (MH " Alcohol Abuse ") or (MH " Alcohol Drinking ") or (MH " Alcoholic Beverages + ") or (MH " Alcoholism ")) and ((MH " Fetus ") or (MH " Pregnancy ") or Prenatal)<br>S3: S1 OR S2<br>S4: (MH " Fetal Alcohol Syndrome/PC ")<br>S5: prevent * or protect * or policy or policies or education or program or health promotion or marketing or best practice * or identifying or identification<br>S6: (S3 AND S5) OR S4<br>S7: Aborig* OR Indig*<br>S8: (MM "Aborigines+") OR (MM "Native Americans") OR (MM "Maori") OR (MM "Indigenous Peoples") OR (MM "Eskimos")<br>Limiters - Language: English<br>S9 S7 OR S8<br>S10 S6 AND S9
Web of Science (ISI Interface)	#1: TI =((fetal alcohol) OR ((pregnan* OR fetus OR prenatal) AND alcohol*)) AND TS=(screening or prevent* or protect* or policy or policies or education or program or health promotion or marketing or identifying OR identification or label*)<br>#2: TS = ((fetal alcohol) OR ((pregnan* OR fetus OR prenatal) AND alcohol*)) AND TI = (screening or prevent* or protect* or policy or policies or education or program or health promotion or marketing or identifying OR identification or label*)<br>#3: #1 OR #2 AND LANGUAGE: (English)<br>#4: TS = (mouse OR mice OR murine OR rat OR rats OR pig OR pigs OR porcine OR sheep) AND LANGUAGE: (English)<br>#5: #3 NOT #4 AND LANGUAGE: (English)<br>#6: TOPIC: (Aborig*)<br>#7: TS= (Aboriginal* OR Indig* OR (First Nation*) OR Native OR (American NEAR/2 Indian) OR Eskimo OR Inuit OR Maori)<br>#8: TI=(Aboriginal* OR Indig* OR (First Nation*) OR Native OR (American NEAR/2 Indian) OR Eskimo OR Inuit OR Maori)<br>#9: #8 OR #7<br>#10: #9 AND #5
PsycINFO (Ovid Interface)	1. exp Fetal Alcohol Syndrome/<br>2. fetal alcohol.tw.<br>3. (alcohol* adj3 (birth defects or congenital malformations)).tw.

	4. fasd.tw.<br>5. fae.tw.<br>6. arbd.tw.<br>7. arnd.tw.<br>8. (alcohol adj3 neurodevelopmental).tw.<br>9. 1 or 2 or 3 or 4 or 5 or 6 or 7 or 8<br>10. alcoholism/ or alcohol abuse/ or alcohol drinking patterns/ or alcohol intoxication/<br>11. exp alcoholic beverages/<br>12. 10 or 11<br>13. pregnancy/<br>14. fetus/<br>15. prenatal exposure/<br>16. 13 or 14 or 15<br>17. 12 and 16<br>18. health education/<br>19. prevention/<br>20. health promotion/<br>21. social marketing/<br>22. health screening/<br>23. screening/<br>24. risk management/ or risk assessment/<br>25. ((reduc* or lower or decrease* or smaller) adj3 risk*).tw.<br>26. prevent*.tw.<br>27. health care policy/ or policy making/ or government policy making/<br>28. protect*.tw.<br>29. 18 or 19 or 20 or 21 or 22 or 23 or 24 or 25 or 26 or 27 or 28<br>30. (9 or 17) and 29<br>31. limit 30 to english language<br>32. limit 31 to animal<br>33. 31 not 32<br>34. exp Indigenous Populations/<br>35. exp American Indians/<br>36. 34 or 35<br>37. 33 and 36<br>38. (Aborig* or Indig*).mp. [mp=title, abstract, heading word, table of contents, key concepts, original title, tests & measures]<br>39. 34 or 35 or 38<br>40. 33 and 39
SocINDEX	S1: fetal alcohol OR fetal alcohol<br>S2: DE “Alcoholism” or DE “Alcoholic Beverages” or DE “Alcoholics” or DE “Drinking of alcoholic beverages”<br>S3: DE “Pregnancy” or DE “Pregnant women” or DE “Teenage pregnancy”<br>S4: S1 OR (S2 AND S3)<br>S5: prevent* or protect* or policy or policies or education or program or health promotion or marketing or best practice or identifying or identification<br>S6: S4 AND S5<br>S7: Aborig* OR Indig*<br>S8: (MM "Aborigines+") OR (MM "Native Americans") OR (MM "Maori") OR (MM "Indigenous Peoples") OR (MM "Eskimos")<br>Limiters - Language: English<br>S9: S7 OR S8<br>S10: S6 AND S9
Informit	(((F?etal alcohol) OR (Alcohol* AND (pregnan* OR *natal OR mother* OR maternal))) AND (Prevent* OR Intervention* OR (Health Promotion) OR (Health Education) OR (Health Communication) OR (Health Plan*) OR (Harm Reduction) OR Treatment* OR Therap* OR Support OR

	Rehab* OR Intervention OR Reduc* OR Abstinence OR Motivation* OR Temperance OR (Alcoholics Anonymous))) AND (Aborigin* OR Indig*)
